# Xylanase supplementation in corn-based swine diets: a review with emphasis on potential mechanisms of action

**DOI:** 10.1093/jas/skaa318

**Published:** 2020-09-24

**Authors:** Amy L Petry, John F Patience

**Affiliations:** 1 Department of Animal Science, Iowa State University, Ames, IA; 2 Iowa Pork Industry Center, Iowa State University, Ames, IA

**Keywords:** arabinoxylan, corn, fiber, pig, xylanase mechanism of action

## Abstract

Corn is a common energy source in pig diets globally; when financially warranted, industrial corn coproducts, such as corn distiller’s dried grains with solubles (**DDGS**), are also employed. The energy provided by corn stems largely from starch, with some contribution from protein, fat, and non-starch polysaccharides (**NSP**). When corn DDGS are used in the diet, it will reduce starch within the diet; increase dietary protein, fat, and NSP levels; and alter the source profile of dietary energy. Arabinoxylans (**AX**s) comprise the majority of NSP in corn and its coproducts. One strategy to mitigate the antinutritive effects of NSP and improve its contribution to energy is by including carbohydrases within the diet. Xylanase is a carbohydrase that targets the β-1,4-glycosidic bonds of AX, releasing a mixture of smaller polysaccharides, oligosaccharides, and pentoses that could potentially be used by the pig. Xylanase is consistently effective in poultry production and moderately consistent in wheat-based swine diets, but its efficacy in corn-based swine diets is quite variable. Xylanase has been shown to improve the digestibility of various components of swine-based diets, but this seldom translates into an improvement in growth performance. Indeed, a review of xylanase literature conducted herein suggests that xylanase improves the digestibility of dietary fiber at least 50% of the time in pigs fed corn-based diets, but only 33% and 26% of the time was there an increase in average daily gain or feed efficiency, respectively. Intriguingly, there has been an abundance of reports proposing xylanase alters intestinal barrier integrity, inflammatory responses, oxidative status, and other health markers in the pig. Notably, xylanase has shown to reduce mortality in both high and low health commercial herds. These inconsistencies in performance metrics, and unexpected health benefits, warrant a greater understanding of the in vivo mechanism(s) of action (**MOA**) of xylanase. While the MOA of xylanase has been postulated considerably in the literature and widely studied in in vitro settings, in wheat-based diets, and in poultry, there is a dearth of understanding of the in vivo MOA in pigs fed corn-based diets. The purpose of this review is to explore the role of xylanase in corn-based swine diets, discuss responses observed when supplemented in diets containing corn-based fiber, suggest potential MOA of xylanase, and identify critical research gaps.

## Introduction

In 2019, more than one billion metric tonne of corn was produced globally with the United States responsible for more than 30% of that supply. Naturally, most of the U.S. swine diets contain corn and many contain corn coproducts, and pig production is concentrated where corn is produced most efficiently. Globally, corn is increasingly used in swine diets, but as with any feed ingredient, it is dependent on price, quality, availability, and accessibility within a region (Popp et al., 2016). Corn is an energy-dense cereal grain with a relatively consistent nutrient composition (NRC, 2012) compared with wheat (Zijlstra et al., 1999) and barley (Fairbairn et al., 1999). Indeed, in the Americas, the economic and nutritional value of other cereal grains is often defined relative to corn. When corn prices are high, often a consequence of turbulent markets, reduced yields, or increased demand for industrial purposes, nutritionists will seek alternatives to reduce feed cost. One such option is industrial coproducts from the dry and wet milling of corn.

A wide array of corn-based industrial coproducts are produced, and corn distiller’s dried grains with solubles **(DDGS**), a byproduct of dry-milled ethanol production, is the most abundant (Shurson et al., 2012). Pigs are fed approximately 10% of the corn DDGS produced annually (Jayasinghe, 2017), and DDGS can effectively supply energy, amino acids, and phosphorus (P). However, one disadvantage of formulating diets containing DDGS, and numerous other industrial coproducts, is their high concentration of non-starch polysaccharides (**NSP**). In recent years, ethanol plants have increased the efficacy with which they extract oil from the solubles fraction, so that swine producers are now utilizing more reduced-oil DDGS. Reduced-oil DDGS contain less fat and greater NSP than conventional DDGS and as such contain less dietary energy (Li et al., 2017). Typically, corn–soybean meal-based diets contain between 7% and 10% neutral detergent fiber (**NDF**), but with every 5% addition of reduced-oil DDGS in place of corn, dietary NDF will increase by approximately 1% (Acosta et al., 2020).

NSP, or more simply called fiber, has taken on many definitions over the past century but has been most recently defined in the context of animal nutritionists by the Codex Alimentarius Committee of the Food and Agriculture Organization of the United Nations (2010) “as those carbohydrate polymers with ten or more monomeric units which are not hydrolyzed by the endogenous enzymes nor absorbed in the small intestine.” Indeed, pigs lack the endogenous carbohydrases needed to digest NSP and rely on microbial fermentation for NSP utilization (Varel and Yen, 1997), and corn-based NSP is poorly fermentable due to its insolubility (Gutierrez et al., 2013). Insoluble NSP is often touted as an antinutritive factor, as increased dietary insoluble NSP will decrease nutrient and energy digestibility, impact pig performance, and reduce carcass yield (Weber et al., 2015; Acosta et al., 2020). However, utilizing exogenous carbohydrases may partially ameliorate these antinutritive effects.

Carbohydrases have gained considerable popularity in the swine industry due to the proven efficacy in pig diets of phytase, another exogenous enzyme that is different in structure but it releases otherwise undigestible phosphorus from phytate. Phytase, as a proof of concept, combined with carbohydrases offering consistent responses when used in poultry diets (Adeola and Cowieson, 2011), supports and encourages this broader enzyme focus. Carbohydrases are enzymes that catalyze the hydrolysis of carbohydrates, and in this context, NSP carbohydrases reduce the molecular weight of NSP by hydrolyzing a targeted type of NSP (Adeola and Cowesion, 2011). Arabinoxylans (**AX**s) comprise nearly half of the NSP found in corn and corn DDGS (Jaworski et al., 2015), and thus, xylanase, a carbohydrase that hydrolyzes the β-1,4-glycosidic bonds of AXs, may mitigate the impact of corn-based NSP. However, the efficacy of xylanases when used in corn-based pig diets is highly variable. Quite often, xylanase will improve the energy and nutrient digestibility of corn-based diets, but this seldom translates into an improvement in growth performance (Torres-Pitarch et al., 2019). Interestingly, increases in markers of improved health and reduced finishing pig mortality have been observed with xylanase supplementation (Zier-Rush et al., 2016).

There has been a large investment in research with xylanase in corn-based diets in the past decade. Still, the responses observed vary considerably, and the in vivo mechanism(s) of action (**MOA**) of xylanase in the presence of corn-based NSP is(are) not yet fully elucidated. This review will explore the role of xylanase in corn-based swine diets, discuss responses observed when xylanase is supplemented in the presence of corn-based fiber, suggest the potential MOA of xylanase, and identify critical gaps in our current knowledge.

## Composition of Corn, Corn Coproducts, and Corn-Based Fiber

To recognize the role of xylanase in corn-based diets, it is pertinent to understand the composition of corn, corn coproducts, and corn-based NSP and to appreciate their influence on diet formulation and gastrointestinal physiology. Corn is a nonviscous cereal grain comprised of approximately 6% bran, 11% germ, and 83% endosperm (Hopkins et al., 1974). The endosperm consists mostly of starch with some structural proteins; the germ contains mainly ether extract; and the bran is predominately NSP (Bach Knudsen, 2014). Accordingly, the nutritional composition of corn is mostly starch, with lower levels of ether extract, protein, and NSP. The composition of corn makes it an excellent source of energy since the metabolic efficiency of glucose, the monomer that comprises starch, is quite high at 0.78 (Patience, 2012). Interestingly, the digestible energy content of corn varies by about 3% (NRC, 2012). This is likely due to the variation in fermentability of NSP among corn samples, even though the contribution of corn NSP to dietary energy is quite small (Petry et al., 2019).

The high starch content in corn explains its attractiveness to ethanol producers; consequently, corn coproducts derived from ethanol production are low in starch (Table 1). Comparatively, coproducts produced from the wet or dry milling of corn differ in their composition and nutritive value, but compared with the parent grain, most of them have increased crude protein (**CP**), fat, minerals, and fiber (Shurson et al., 2012). Discussing the nutrient composition among the vast array of corn coproducts is outside the scope of this review—readers are referred to Zijlstra and Beltrana 2013) and Gutierrez et al. (2014)—and discussion will be limited to corn DDGS because it is by far the most common corn coproduct used in practical pig feeding programs. Within DDGS, there is considerable compositional variability. For example, Zeng et al. (2017) reported that the coefficients of variation for CP (*n* = 90), NDF (*n* = 90), ash (*n* = 46), and crude fat (*n* = 38) in corn DDGS were 9%, 13%, 25%, and 36%, respectively. This is likely a result of the processes used to extract ethanol by a given plant and not necessarily the region in which the DDGS is sourced (Stein et al., 2009; Pedersen et al., 2014). Nonetheless, in general, reduced-oil DDGS contains 210%, 234%, 235%, and 156% more ash, NDF, CP, and ether extract than corn, respectively, and five times less starch (NRC, 2012).

**Table 1.** Total NSP, NDF, ADF, TDF, starch, and AX concentrations (g/kg) in corn-based feed ingredients

Feed ingredient	Total NSP	NDF	ADF	TDF	Starch	AX^1^	A:X^2^
Corn^3,4,5^	81	85	24	108	625	38	0.81
Dehulled, degermed corn^3^	11	38	4	23	685	8	1.00
Corn gluten meal^3^	49	121	70	88	120	20	1.22
Corn bran^3^	370	406	105	425	211	221	0.56
Corn bran with solubles^3^	171	227	51	253	190	95	0.58
Cooked Corn DDGS^3^	204	345	92	326	28	108	0.83
Corn DDGS–reduced oil^3^	250	387	143	329	29	143	0.79
Uncooked DDGS^3^	220	308	79	291	52	113	0.77
High protein DDG^3^	219	311	118	289	82	92	0.80
Corn germ meal^3^	444	462	115	441	164	292	1.18
Corn gluten feed^5,6^	287	275	84	316	111	145	0.69

^1^Arabinose + xylose from total NSP analysis.

^2^Ratio of arabinose to xylose.

^3^Adapted from Gutierrez et al. (2014).

^4^Adapted from Navarro et al. (2018).

^5^Adapted from Jaworski et al. (2015).

^6^Adapted from NRC (2012).

**Figure 1.**
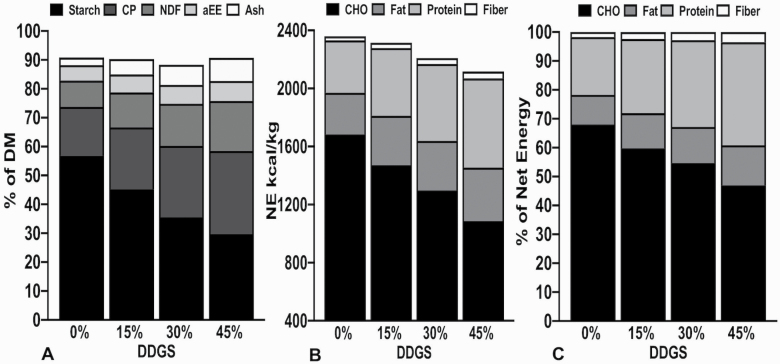
The impact of substituting reduced-oil DDGS at the expense of corn using a constant ingredient formulation on nutrient composition (A), NE (B), and contribution of simple carbohydrates, fat, protein, and fiber to NE (C).

When reduced-oil DDGS are included in diet formulations at the expense of corn, the nutrient composition will change along with net energy (**NE**; Patience and Petry, 2019). This can be visualized by estimating the NE content and composition of NE of the constant ingredient formulation in a study by Acosta et al. (2020). In this study, for every 15% increase in reduced-oil-corn DDGS included at the expense of corn, dietary acid-hydrolyzed ether extract increased by nearly 17%, NDF increased by 34.2%, CP increased by 27%, starch decreased by 20% (Figure 1A), and estimated NE decreased by 83 kcal/kg or 3.5% (Figure 1B). Similarly, with increasing levels of reduced-oil DDGS, the contribution of fat, fiber, and protein to NE will increase, and the contribution of simple carbohydrates will decrease (Figure 1C). Furthermore, when these components are regressed in relationship to increasing levels of reduced-oil DDGS, for every 1% of corn replaced by reduced-oil corn DDGS, the amount of NE coming from fat, CP, and fiber increases by 0.1%, 0.3%, 0.02%, respectively, but the amount of NE coming from simple carbohydrates decreases by 0.44%.

The pig will utilize these four components—fat, CP, fiber, and starch—for energy with varying metabolic efficiencies, and it is well supported that the least efficient of them is fiber. The fermentation of NSP contributes about 30% less energy compared with enzymatically digested carbohydrates (Noblet and Le Goff, 2001; Patience, 2012). In Figure 1C, there is a nearly 3-fold increase in the contribution of fiber to energy from 0% DDGS inclusion to 45%, but the relative contribution of fiber to energy is quite small, less than 5%. These inefficiencies are largely attributed to the increased energetic cost of ingestion, digestion, fermentation, and metabolism of NSP, and the subsequent effects of fiber on the maintenance energy requirement of the pig (Noblet and Le Goff, 2001; Agyekum and Nyachoti, 2017). Even the slightest alteration in dietary energy can be costly to a producer, as it affects nearly every performance metric, and meeting the specification for dietary energy accounts for more than 60% of the input cost of raising one pig to market (Beaulieu et al., 2009; Patience, 2017). Thus, if xylanase can improve the overall contribution of corn-based fiber to NE, it would add considerable value to the swine industry. Moreover, if xylanase promotes the intestinal absorption of individual monosaccharides that constitute fiber, rather than microbial fermentation, it could improve the metabolic efficiency by which the pig utilizes fiber for energy.

In addition to fiber source, the type and concentration of fiber within a diet need to be considered when using exogenous carbohydrases. Due to the complexity of the chemistry of dietary fiber, in practical terms, it is defined by the analytical method used to quantify it. There are several methods for analyzing dietary fiber, and the total NSP, NDF, acid detergent fiber (**ADF**), and total dietary fiber (**TDF**) content of corn and 11 corn coproducts have been summarized in Table 1. Of these fiber analyses, TDF is the most encompassing, and arguably the most accurate, as it measures both insoluble and soluble dietary fibers (**SDF**; Fahey et al., 2019). However, the SDF concentration of corn is less than 0.5%, and in corn coproducts, it is generally less than 1.5% (Navarro et al., 2018; Abelilla and Stein, 2019). As such, the detergent system (NDF and ADF) created by Van Soest (1963) is a suitable indicator of the fiber concentration of corn and corn coproducts as it measures the insoluble fiber fraction, but there are analytical limitations (Fahey et al., 2019). This is apparent when comparing the NDF and TDF values in Table 1. The detergent system does not capture SDF, and in the TDF analysis, an analyte is corrected for ash and protein residues, while NDF is not. This is likely why NDF is higher than TDF in the coproducts with higher protein and ash concentrations. The total NSP analysis proposed by Englyst et al. (1994) gives insight into fiber types within a feed ingredient, when the individual monomeric components are measured using chromatography, but this methodology consistently underestimates fiber, relative to TDF, and is not a methodology approved by the Association of Official Analytical Collaboration (AOAC) International.

Corn DDGS has nearly three times the TDF and NDF content of corn, and corn bran and corn germ meal almost four times (Table 1); not surprisingly, the composition of that fiber is quite similar. The NSP in corn and corn DDGS is composed of approximately 49% AXs, 22% to 23% cellulose, and the remaining 28% to 30% is a mixture of other hemicelluloses, such as mixed β-glucans and β-mannans (Bach Knudsen, 2014; Jaworski et al., 2015). Although less than 2% of the total NSP composition, the β-mannan content in corn DDGS is substantially greater than corn, and this is likely a result of the presence of residual yeast from ethanol production (Pedersen et al., 2014; Shurson, 2018). Corn also contains about 1.5% resistant starch that can be rapidly fermented in the large intestine of the pig (Bird et al., 2007); corn DDGS contains between 3% and 14% residual starch that escapes fermentation to ethanol and is mostly resistant starch types 1 and 2 (Li et al., 2014; Pedersen et al., 2014).

These NSP also have viscosity, hydration, and cation exchange properties (Kritchevsky, 1988). They can influence gastrointestinal physiology by altering digesta transit time, increasing endogenous losses, modulating microbial activity, reducing nutrient digestibility, and impeding nutrient uptake (Blackwood et al., 2000). Relative to sugar beet pulp, a feed ingredient with 3 to 4 times the amount of SDF, corn and corn DDGS have less swelling and water-binding capacity, and lower viscosity (Navarro et al., 2018). Moreover, due to its low solubility, corn NSP possesses invariant exchange properties that can hinder calcium uptake in the small intestine (Laszlo et al., 1992). However, physicochemical properties may not always be indicative of a given physiological response (Gidley and Yakubov, 2019). For example, it is often stated that insoluble NSP is poorly fermentable; while this is the case for corn-based feedstuffs, NSP stemming from pea hulls are insoluble, but readily fermentable (Jha and Leterme, 2012; Gutierrez et al., 2013). If xylanase hydrolyzes corn NSP, it could potentially alter its physicochemical properties and as such may mitigate its impact on digestive physiology. The attenuation of digesta viscosity from feeding viscous cereal grains is the hallmark of the success of xylanase in the poultry industry (Raza et al., 2019), but its efficacy in viscous swine diets is less consistent (Patience et al., 1992; Lærke et al., 2015).

Xylans are the target substrate of xylanase. They are composed of a d-xylose backbone linked by β-1,4-glycosidic bonds in a linear or branched form and are frequently substituted with other monosaccharides, phenolic compounds, and short-chain fatty acids (Cummings and Stephen, 2007). AX is the dominant xylan found in corn and corn coproducts, with the majority being concentrated in the bran and far less in the endosperm. This is apparent when comparing the AX content in dehulled and degermed corn (8 g/kg) vs. corn bran (221 g/kg), and logically, corn-based feed ingredients with increased NDF and TDF have increased AX concentrations (Table 1). There is certainly an opportunity to improve the utilization of AX from corn; for example, Gutierrez et al. (2014) concluded that across nine corn coproducts, their AX concentration best explained the variance in the apparent ileal digestibility (**AID**) of gross energy (**GE**) and dry matter (**DM**), and NSP xylose concentration also best explained the variance in the apparent total tract digestibility (**ATTD**) of GE, DM, and NDF.

As the name suggests, AXs are highly substituted with l-arabinose, and up to 85% of the xylose monomers in corn bran are substituted with various residues and the majority are arabinose side chains linked by α-1,2- or α-1,3-glycosidic bonds (Chanliaud et al., 1995). Additionally, galactose and glucuronic acid can form glycosidic bonds to the xylose backbone, and acetic acid can also directly esterify to the main chain of corn-based AXs and accounts for approximately 4% of the dry weight of corn bran (Saulnier et al., 1995). To complicate things further, almost 40% of the arabinose substitutions in corn-based AXs are nonterminal, and contain glycosidic linkages to other arabinoses, xyloses, or galactose units, or are esterified to hydrocinnamic acids (Chanliaud et al., 1995). The most commonly substituted hydrocinnamic acid in corn-based AXs is ferulic acid; *p*-coumaric is also present but to a lesser degree (Saulnier et al., 1995). Moreover, corn-based AXs can be cross-linked through dehydrodiferulate or dehydrotriferulate ester bridges formed between the arabinose–ferulic acid complexes (Saulnier et al., 1995).

These aforementioned structural complexities contribute to the insolubility of corn-based AXs within the gastrointestinal tract, but their fermentability is not necessarily dependent on solubility. However, due to the degree of interaction of corn AXs with other plant components, increased phenolic cross-linkages, arabinose substitutions, and lignification, it is also poorly fermented by resident microbiota (Bach Knudsen, 2014). The ratio of arabinose to xylose (**A:X**) is used as an indicator of AX solubility, as a higher A:X would indicate greater arabinose substitution. For example, compared with corn, the AXs in wheat and rye are considerably more soluble and viscous, and their A:X ratio ranges between 0.57 and 0.7 (Lærke et al., 2015; Buksa et al., 2016), whereas the A:X ratio for corn is 0.81 (Table 1). Similarly, the A:X of wheat DDGS is 0.68, and corn DDGS ranges between 0.77 and 0.83 (Pedersen et al., 2014; Table 1). Interestingly, though, the ratio of A:X in corn bran is similar to that of rye and wheat, yet the fermentation of corn bran is quite poor in the pig (Gutierrez et al., 2013). This is likely due to the increased lignification in corn bran (Akin and Rigsby, 2008). Interestingly, corn bran appears to be susceptible to xylanase in the pig (Petry et al., 2020b). Moreover, the structure of AX is often described and portrayed in a linear unidimensional fashion, but the reality is the more substituted and complex AXs, like those in corn, form complex three-dimensional structures that are intertwined with other components in the plant cell wall (Jeremic et al., 2014). In total, these structural complexities pose challenges with utilizing exogenous xylanase, but nevertheless, reports of positive responses exist when xylanase is supplemented in diets containing corn-based fiber (Torres-Pitarch et al., 2019).

## Xylanase and Corn-Based Fiber

The complete saccharification of corn-based AX requires nine enzymes. Endoxylanases (i.e., xylanase) are needed to hydrolyze the β-1,4-glycosidic bonds of the main chain; several de-branching enzymes, such as α-arabinofuranosidases, α-arabinofuranohydrolases, α-glucuronidases, α-galacturonidases, and acetyl xylan esterases, are needed to hydrolyze the side chains; β-d-xylosidases are needed to release xylose for the various oligomers produced; and ferulic and *p*-coumaric acid esterases are required to release phenolics esterified to arabinose (Dodd and Cann, 2009; Stein, 2019). However, the complete in vivo saccharification of AX is likely implausible, nor worth the return on investment, due to the vast array of technologies and advancements needed to produce these enzymes in sufficient quantities with proven efficacies to tolerate the conditions of the gastrointestinal tract (Bedford, 2019). Nonetheless, some of these “accessory” enzymes are found in commercially available xylanases, as many are produced from the various microorganisms that produce xylanase, but by and large, endoxylanase is the most readily available commercial carbohydrase that targets AXs.

Xylanase is a glycoside hydrolase (**GH**), and of the 166 GH characterized, 9 GH families are associated with xylanases, 6 of which have a single catalytic domain and 3 have two catalytic domains (Carbohydrate Active Enzymes Database; Lombard et al., 2014). The majority of xylanase research in animal nutrition has focused on GH families 10 and 11, which possess single catalytic domains. Pedersen et al. (2015) found that GH 10 xylanases are more efficient in degrading corn DDGS AX than GH 11. Xylanase is produced by a variety of microorganisms, and commercially available exogenous xylanases are predominantly produced from bacteria or filamentous fungi (Bhardwaj et al., 2019). There is a dearth of studies comparing the efficacy of varying sources and types of xylanase when supplemented with corn-based fiber in the pig. However, a study by Ndou et al. (2015) evaluated five mono-component xylanases from different microbial origins and preparations in a diet composed of corn and corn DDGS. The authors reported that xylanase from *Fusarium verticilloides* improved average daily gain (**ADG**) and feed efficiency (**G:F**) but they found no improvements in growth performance when using xylanase prepared from *Aspergillus clavatus*, *Bacillus subtilis*, or two from *Trichoderma reesei*. Interestingly, Ndou et al. (2015), in a separate digestibility experiment, observed improvements in the AID of NSP and AX from all xylanase sources except *Bacillus subtilis.*

### The paradigm among digestibility, growth performance, and health responses in pigs fed xylanase

The absence of a performance response but improved fiber digestibility observed by Ndou et al. (2015) is not abnormal (Passos et al., 2015; Yang et al., 2016). In theory, supplementing xylanase in corn-based swine diets should improve nutrient and energy digestibility, and subsequently growth performance (Patience, 2012). A meta-analysis of 67 studies reflecting a variety of basal diet composition conducted by Torres-Pitarch et al. (2019) concluded that irrespective of diet composition, xylanase improved the ATTD of DM, GE, and CP, but growth performance responses were not observed as frequently. For the purposes of this review, a targeted literature search was conducted using Google Scholar and EBSCO from January 2002 to July 2020 for digestibility and growth performance studies that met the following criteria:

- Results were published in a non-predatory peer-reviewed journal;
- The study evaluated xylanase as the sole carbohydrase in at least one dietary treatment;
- Corn grain and/or corn coproducts were the only cereal source within the diet;
- The study included a control diet of similar formulation to the diet with xylanase.

**Figure 2.**
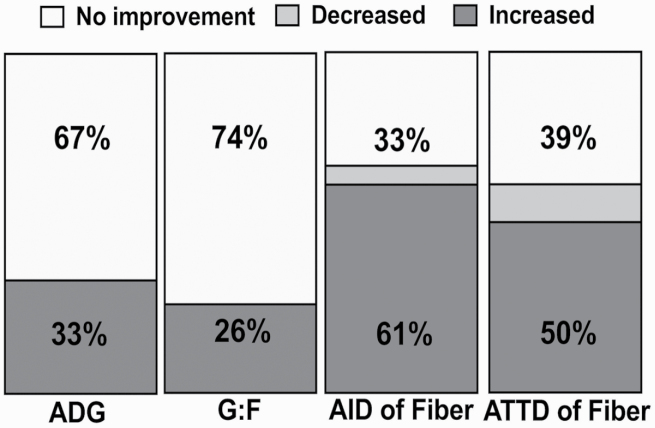
The efficacy of xylanase to improve ADG, G:F, AID of fiber (NSP, TDF, or NDF), and ATTD of fiber of pigs fed corn-based diets from 2000 to July 2020.

A total of 19 publications met the search criteria. Among the growth performance observations within these studies, in 33% and 26% of the studies, xylanase improved ADG and G:F, respectively, but no improvements in average daily feed intake were reported (Figure 2). Xylanase improved the AID and ATTD of “fiber” (NSP, TDF, or NDF) by 61% and 50% of the time, respectively. It should also be noted that this search would be biased due to the difficulty in publishing data that conclude no treatment effect. There is a lack of empirical understanding on why this dearth of performance responses exists, nor are there any obvious trends which explain those publications reporting improved performance vs. those that did not. 

A variety of experimental designs, diet formulation strategies, enzyme doses, xylanase sources, fiber analyses, sample sizes, and stages of production existed among these studies. However, if one looks past this variability, there is some evidence to suggest that adaptation time may play a role in xylanase efficacy. A study by Petry et al. (2020b) found that xylanase improved ADG and G:F in pigs fed a corn-based diet with 30% corn bran without solubles after 14 d of supplementation, and these metrics were even greater after day 27. This work is in agreement with Fang et al. (2007), Myers and Patience (2014), and Lan et al. (2017) who found that xylanase improved performance in later phases of supplementation in pigs fed largely corn-based diets. Conversely, work by Ndou et al. (2015) and Kerr et al. (2013) observed no improvements in performance among seven xylanase sources supplemented for greater than 30 d. This variability in both methods and responses among these studies indicates that there is a need for more robust and standardized evaluation of exogenous enzymes, much like what has been proposed for the study of alternatives to antibiotic growth promoters (Olsen et al., 2018).

Although more than half of the studies reported an improvement in fiber digestibility, when compared with studies using wheat-based diets, digestibility responses in pigs fed corn-based diets are still less common (Torres-Pitarch et al., 2019). When xylanase is ineffective, it is often suggested that corn AX is recalcitrant to enzymatic hydrolysis because of its structural complexity and subsequent insolubility. However, the number of studies that report improved fiber digestibility and the magnitude of those responses certainly indicate that xylanase is likely hydrolyzing fiber directly or indirectly through microbiota modulation. Moreover, there is considerable in vitro work that confirms xylanase’s ability to hydrolyze corn-based AXs into soluble, more fermentable fractions (Pedersen et al., 2015; Kiarie et al., 2016), but in vitro conditions may not always translate well to that which exists in vivo. Similar to the performance observations, enzyme adaptation may play a role in the efficacy of xylanase to improve digestibility, particularly in the upper small intestine. A recent study suggested that 25 d of adaptation was required for xylanase to improve fiber and energy digestibility in the upper small intestine (medial jejunum—292 ± 12 cm distal to the pyloric sphincter) of growing pigs fed insoluble corn fiber; in contrast, responses across the total gastrointestinal tract were observed after only 7 d of adaptation (Petry et al., 2020a). Perhaps, the adaptation of the upper small intestine to AX hydrolysis may partially explain the discrepancies between performance and digestibility responses previously discussed. Digestibility studies often employ adaptation periods of less than 10 d, and minimal research has been conducted in the upper small intestine. Further research is clearly warranted to understand the role of adaptation and xylanase efficacy in corn-based diets.

While the original intent of using xylanase was to improve performance through increasing fiber utilization, today an increasingly common justification is its ability to reduce grow-finish pig mortality. It has been reported that supplementing xylanase can increase the net live margin per pig started by up to US$2.51 in one production system, largely due to the improvements in mortality (Zier-Rush et al., 2016). Reductions in mortality have also been observed among several commercially available xylanases in different production systems with varying health statuses and diet compositions (Zier-Rush et al., 2016; Beckers, 2017). Economically, relative to improving G:F by 2 points, reducing wean-to-finish mortality from 4% to 2.4%, as observed by Zier-Rush et al. (2016), is nearly four times more profitable. Moreover, recent research in corn-based diets has suggested that xylanase may improve pig health through altering intestinal morphology (Duarte et al., 2019; Li et al., 2019; Chen et al., 2020), increasing gut barrier integrity (Tiwari et al., 2018; Petry et al., 2020b), altering immune function (Chen et al., 2020), and mitigating localized and systemic oxidative stress (Duarte et al., 2019; Petry et al., 2020b). The efficacy of xylanase to improve pig health and reduce mortality is clearly an added, and until recently unexpected, benefit of using this enzyme. As the pork industry transitions to reduce in-feed antibiotic usage, the role of xylanase in this regard may be enhanced (Melo-Duràn et al., 2019). Further, it is apparent that understanding the in vivo MOA of xylanase is imperative to eliciting more consistent and synergistic responses in pig production, understanding its true role in diet formulation, and maximizing its efficacy in U.S. swine diets.

## The MOA of Xylanase in Pigs

### Xylanase releases monosaccharides and oligosaccharides

Logically, xylanase likely hydrolyzes AX into lower molecular weight fragments that can be either absorbed or fermented in the gut (Adeola and Cowieson, 2011). Certainly, the improvements in fiber digestibility imply this, particularly the digestibility of AX, but the direct quantification of these products has not been conducted in pigs fed corn-based diets. Recent work by Tiwari et al. (2018) suggests that xylanase supplemented in a corn-based diet increased soluble NSP production in the ileum, and this is possibly a result of soluble AX fragments released from the insoluble AXs found in corn (Pedersen et al., 2015). However, the composition of these soluble AX fragments, and the efficiency by which the pig utilizes them, remains poorly understood.

Characterizing the release products of xylanase, in vivo, is enormously challenging both analytically and from an experimental design perspective. Arabino-xylooligosaccharides (**AXOS**) with varying degrees of polymerization are the most plausible release products of xylanase, and this has been confirmed in pigs fed wheat, rye, and wheat DDGS (Laerke et al., 2015; Pedersen et al., 2015). However, there are a plethora of potential release products, depending on the composition of the initial AX substrate; because of the aforementioned complexity of corn-based AX, the potential oligomers produced in vivo are exponentially more complex and difficult to isolate. The less substituted oligomers can be semiquantitatively characterized using various chromatographic techniques, and these methodologies are advancing considerably due to the interest in prebiotics among various industries (Alyassin and Campbell, 2019). It is plausible that in the near future, these analyses will be more routine and quantitative in nature, thus allowing insight into what is released by xylanase in vivo. In addition to the analytical challenges, many of the potential release products are rapidly metabolized by gastrointestinal microbiota, and, as such, understanding microbial metabolites could be pertinent to understanding the in vivo release products of xylanase (Feng et al., 2018).

Differentiating the composition of the released products from xylanase is imperative to determining the efficiency by which the pig utilizes them for energy. Generally, energy derived from carbohydrate fermentation is metabolically less efficient relative to the direct metabolism of monosaccharides, and this is certainly the case for glucose. Conversely, it is unclear if the energetic efficiency of xylose would be greater if fermented (Huntley and Patience, 2018; Abelilla and Stein, 2019). A study by Laerke et al. (2015) found that xylanase increased the xylose concentration in the liquid phase of ileal digesta from pigs fed wheat by nearly 4-fold, but not in pigs fed rye. Moreover, an increase in urine GE in pigs fed corn bran with xylanase supplementation was observed by Petry et al. (2020b); this is perhaps indicative of xylose absorption as it has been reported that free xylose increases urine GE due to the excretion of xylose itself or threitol (Huntley and Patience, 2018). There is a dearth of in vivo evidence to support xylose release from corn-based AX by xylanase, and in vitro research would suggest that it is quite marginal (Dale et al., 2019). This lack of discernment in xylanase’s release products is a bottleneck for fully elucidating the MOA of xylanase in pigs fed corn-based fiber and could aid in understanding the commonly observed misalignment between digestibility and performance. However, the plausibility of xylanase hydrolyzing corn-based AX to some degree is logical and well supported by in vitro research. Moreover, the subsequently discussed MOAs are dependent on xylanase hydrolyzing AX to some degree.

### Xylanase mitigates the impact of NSP physicochemical properties

It is well established in poultry that xylanase mitigates the impact of viscous cereal grains on the viscosity of intestinal contents by hydrolyzing soluble AXs (Raza et al., 2019). Viscous polysaccharides form gels in intestinal digesta, consequentially increasing digesta viscosity and reducing nutrient digestibility by decreasing lipid emulsification and preventing the interaction of nutrients with the intestinal brush border (Dikeman and Fahey, 2006). However, corn-based diets are largely insoluble and nonviscous and as such likely have a limited impact on digesta viscosity (Navarro et al., 2018). Moreover, work by Hooda et al. (2011) using both high- and low-viscosity diets suggested a moderately positive relationship between increased digesta viscosity and the AID of GE in pigs, likely due to increased mean retention time, and lower viscosity in pigs (Lee et al., 2010; He et al., 2020). Potentially, the impact of digesta viscosity on nutrient digestibility is less pertinent in swine compared with poultry, and even more so in diets with nonviscous feed ingredients.

However, there is evidence to imply that xylanase does reduce jejunal digesta viscosity in pigs fed corn-based diets (Tiwari et al., 2018; Duarte et al., 2019; Chen et al., 2020), but others have found no impact on jejunal digesta viscosity (He et al., 2020). Moreover, a quadratic relationship on stomach and jejunal digesta with increasing xylanase supplementation levels in corn-based diets has been observed (Passos et al., 2015; He et al., 2020). These studies demonstrated at intermediate levels of supplementation that the viscosity of digesta is decreased, but at greater levels, it is increased, relative to no inclusion. Perhaps, increased soluble NSP production from xylanase hydrolysis of insoluble corn-based AXs may have indirectly increased viscosity at a greater supplementation level. Moreover, it should be noted that these studies measured the viscosity of the digesta supernatant and only at one or two shear rates. Intestinal digesta displays pseudoplastic shear thinning behavior, and as such, when the shear rate of the viscometer increases, viscosity decreases (Dikeman and Fahey, 2006). Therefore, measuring digesta viscosity at one or two shear rates may inadequately portray the influence of xylanase. Similarly, measuring the viscosity of digesta supernatant is not directly indicative of the rheological properties of whole digesta. Research by Takahashi and Sakata (2004) suggested that the solid particles in pig cecal digesta are largely responsible for their rheological properties. Further research is warranted to determine the impact of xylanase on the rheological properties of whole digesta from pigs fed corn-based fiber.

### Xylanase releases trapped nutrients

AXs are an integral component of the plant cell-wall structure, and particularly in cereal grains, they create architecture around starch and protein granules within the aleurone. This is often referred to as the nutrient encapsulation or caging effect of NSP. One conceivable MOA of xylanase is that it can disrupt this structural architecture, through AX hydrolyzation, and, in turn, expose stored starch and protein granules to endogenous enzymes and microbial fermentation (Bedford and Schulze, 1998; De Lange et al., 2010). The improvements in the digestibility of unexpected dietary components, such as starch, CP, and various minerals and amino acids, with xylanase supplementation support this MOA. However, these digestibility responses are inconsistent in pigs fed corn-based diets (Kerr et al., 2013; Passos et al., 2015), and digestibility metrics provide no discernment if it is due to the enzyme or modulation of the microbiome. Conversely, there is considerable in vitro microscopic evidence to support this MOA (Le et al., 2013; Jha et al., 2015; Ravn et al., 2016), but minimal work has been conducted in corn-based feed ingredients or in vivo. Indeed, the nutritional and economic value of xylanase is amplified if the caging effect of NSP can be consistently mitigated in vivo; further research is needed to confirm this MOA in corn-based diets. Where this mitigation occurs is also of importance, as released starch is of more value if released in the small intestine, and amino acids released in the hindgut serve little value to pig. In the future for swine nutritionists to have confidence in a potential nutrient release value of xylanase, considerable quantitative research is needed in this area. Employing more advanced quantitative microscopy methodologies, such as those described by Bourlieu et al. (2020), would be warranted. However, relative to the economics of reducing mortality, pursuing a nutrient matrix release value for xylanase would likely provide minimal to no return on investment due to the capital-intensive research required and marginal payoff in terms of increased nutrient availability.

### Xylanase modulates gastrointestinal microbiota to improve pig health

Recently, there has been an increase in the evidence supporting xylanase supplementation for purposes that extend beyond the logical improvement in fiber utilization. The aforementioned reductions in mortality are one of the more consistent responses observed commercially with this enzyme, and recent research suggests that xylanase likely modulates intestinal health through mechanisms that improve gut barrier integrity, mitigate oxidative stress, and alter immune function (Li et al., 2018; Duarte et al., 2019; Petry et al., 2020b). These improvements observed in the systemic and gut health of the pig are most plausibly a result of the interplay between xylanase and the microbiome. Moreover, with the recent improvements in both availability and cost of culture-independent next-generation sequencing technologies, the interplay between the microbiome and nutrition has gained greater importance. The MOA for how xylanase modulates gastrointestinal microbiota is multifactorial, and the magnitude of microbial modulation differs among these MOA.

Certainly, the improvements in the AID of nutrients, through either the mitigation of increased digesta viscosity or the caging effect of NSP, can have an impact on gastrointestinal microbiota. Through these MOA, xylanase could decrease the quantity of undigested substrates in ileal digesta and as such alter substrate composition accessible to the large intestine microbiota. Thus, xylanase may partially ameliorate substrate competition among the host and microbiota and potentially reduce the colonization of pathogenic bacteria through microbial starvation (Bedford and Cowieson, 2012). Moreover, if protein fermentation is partially mitigated through increased utilization in the upper gut, this would be beneficial by reducing the production of branched-chain fatty acids and protein fermentation products, both of which can hinder microbial diversity (Peng et al., 2017). In contrast, if xylanase improves protein accessibility in the hindgut through the nutrient release MOA, it has the potential to exacerbate the effects of protein fermentation. However, the more plausible and direct MOA for modulation of the microbiome is the “prebiotic” or “stimbiotic” effect of the released AXOS from the hydrolyzation of AX. In situ AXOS production has the potential to modulate gastrointestinal microbiota through exerting prebiotic-like effects in the gut that improves intestinal barrier function and integrity and invokes an immunostimulatory response by the gut-associated lymphoid tissue (Tiwari et al., 2020). Moreover, these oligomers can act as a stimbiotic that stimulates luminal conditions to favor the proliferation of microbiota that degrade AX more efficiently and are generally “beneficial” in nature (Bedford, 2018; Bautil et al., 2020).

If xylanase is effective in modulating microbial ecology to favor corn-based AX fermentation, this would be beneficial not only in terms of capturing a greater amount of energy from a highly insoluble and poorly fermentable fiber source but also potentially improving gastrointestinal health through the stimulatory effects of short-chain fatty acids (Bach Knudsen et al., 2018). Indeed, a carbohydrase blend containing xylanase demonstrated positive effects in controlling postweaning colibacillosis and modulating the microbiome (Li et al., 2019, 2020). A recent study by Luise et al. (2020) observed that xylanase tended to increase jejunal villi height and upregulate *Lactobacillus reuteri* in nursery pigs challenged with enterotoxigenic *Escherichia coli* and fed corn-based diets. This MOA could also partially explain why xylanase improved diversity and altered microbiota ecology in the large intestine of pigs fed corn-based feed ingredients (Zhang et al., 2017, 2018). Likewise, the observations that xylanase mitigates oxidative stress in pigs fed corn-based diets observed by Duarte et al. (2019) and Petry et al. (2020b) may be partially explained through this MOA. As discussed previously, corn AX is highly substituted with ferulic acid, and this phenolic compound is a robust antioxidant that is efficient in mitigating free radicals, increasing anti-oxidase production, and hindering enzymes that produce excess free radicals (Ogiwara et al., 2002). However, ferulic acid bioavailability is low, but in vitro evidence suggests that xylanase can improve its bioavailability by releasing feruloylated AXOS, and, in turn, ferulic acid could be released by microbial ferulic acid esterase (Mathew and Abraham, 2004). Still, this has yet to be confirmed in vivo in the pig. However, these improvements in the various aspects of gastrointestinal structure and function warrant further investigation into this MOA. Moreover, there is a paucity of studies investigating gastrointestinal microbiota composition and phenotypic responses in pigs fed corn-based diets.

## Future Considerations and Conclusions

Supplementing xylanase emerged in an effort to ameliorate the antinutritional effects of NSP, and in corn-based swine diets, there is certainly ample substrate and opportunity to improve the energetic contribution of fiber. However, its efficacy in improving fiber utilization and pig performance is variable and still poorly understood. Interestingly though, unexpected health benefits are often observed when including xylanase in diet formulations. Indeed, there have been considerable advancements in understanding why a fiber-degrading enzyme could improve pig livability, but there is little empirical understanding of why digestibility and performance responses are misaligned. Even a brief review of the literature would indicate this may be a product of variable experimental conditions and designs and indicate there is a need for more robust and standardized research protocols in carbohydrase research. Recently, adaptation time has emerged as an experimental design criterion, which, if proven to be important, could possibly improve research outcomes. Although research on adaptation time is in its infancy, there is considerable evidence to suggest that previous studies could have faltered due to inadequate adaptation periods.

Moreover, it is increasingly apparent that elucidating the in vivo MOA of xylanase in pigs fed corn-based swine diets is warranted to improve its use in swine diets. If the MOA is correctly determined and robustly tested, swine nutritionists will be able to make informed decisions about the proper utilization of xylanase and will be more confident in achieving consistent and economically valuable phenotypic outcomes. By the same token, the desired phenotypic outcome from xylanase may need to be reevaluated. Certainly, an improvement in ADG or G:F can be valuable to a producer, but as it stands, these responses are not yet consistent in commercial situations. However, improving pig livability appears to be an unexpected benefit. Economically, there is certainly an upside to improving the proportion of full value pigs, if market conditions favorite it.

Several potential MOA for xylanase have been proposed over the years, and in the context of corn-based diets, several are plausible, and as with many feed additives, it is likely multifactorial. The modulation of the microbiome through a stimbiotic mechanism, while the least studied, has the potential to provide the greatest return on investment. The mitigation of increased digesta viscosity by xylanase likely has the least value and plausibility as corn-based diets are nonvicious and highly insoluble. Partially ameliorating the nutrient encapsulation of NSP through xylanase supplementation is certainly well supported by in vitro evidence and reported improvements in the digestibility of unexpected dietary components, but the monetary investment required to apply a nutrient release value to xylanase is likely not worth the marginal improvements observed. All of the aforementioned MOA rely on xylanase hydrolysis of AX to some extent. While there is certainly logical evidence that this is occurring in vivo, the composition of associated release products is largely unknown. All of this is essential to our understanding of the *in vivo* MOA of xylanase, including how these breakdown products’ contribution energy to the pig. Further, the chemical complexity of corn-based AXs hinders our ability to isolate these release products and quantify them. Recent advances in chromatographic methodologies provide optimism that they will be characterized, and in the future, this could become a routine analysis. Continual holistic and multifaceted investigation into the MOA of xylanase in corn-based diets will exponentially improve our understanding of enzymes and probably stimulate the increased use of carbohydrases in swine diets.

## Abbreviations

ADF: acid detergent fiber
ADG: average daily gain
AID: apparent ileal digestibility
ATTD: apparent total tract digestibility
AX: arabinoxylans
A:X: ratio of arabinose to xylose
AXOS: arabino-xylooligosaccharides
CP: crude protein
DDGS: distillers’ dried grains with solubles
DM: dry matter
GE: gross energy
G:F: feed efficiency
GH: glycoside hydrolase
MOA: mechanism(s) of action
NDF: neutral detergent fiber
NE: net energy
NSP: non-starch polysaccharides
SDF: soluble dietary fiber
TDF: total dietary fiber
